# Supplementary material for: Polymer Thin Film Promotes Tumor Spheroid Formation via JAK2-STAT3 Signaling Primed by Fibronectin-Integrin α5 and Sustained by LMO2-LDB1 Complex
Source: Biomedicines. 2022 Oct 24;10(11):2684. doi: 10.3390/biomedicines10112684 (PMC9687151; doi:10.3390/biomedicines10112684)
Supplement: Supplementary file 1 [file biomedicines-10-02684-s001.zip › biomedicines-1954646 Supplementary Figure.pdf]

Supplementary Figures

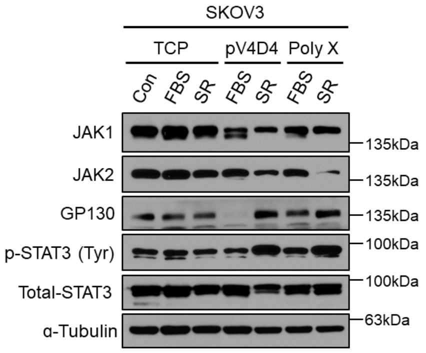

Figure S1. Expression of GP130-JAK-STAT3 signaling proteins in the SKOV3 cells grown under polymer X, pV4D4, or TCP culture conditions supplemented with FBS or SR. Cell lysates from SKOV3 cancer cells cultured on TCP or pV4D4 or polymer X by date were immunoblotted with antibodies specific to JAK1, JAK2, GP130, p-STAT3, total STAT3, and α-tubulin.

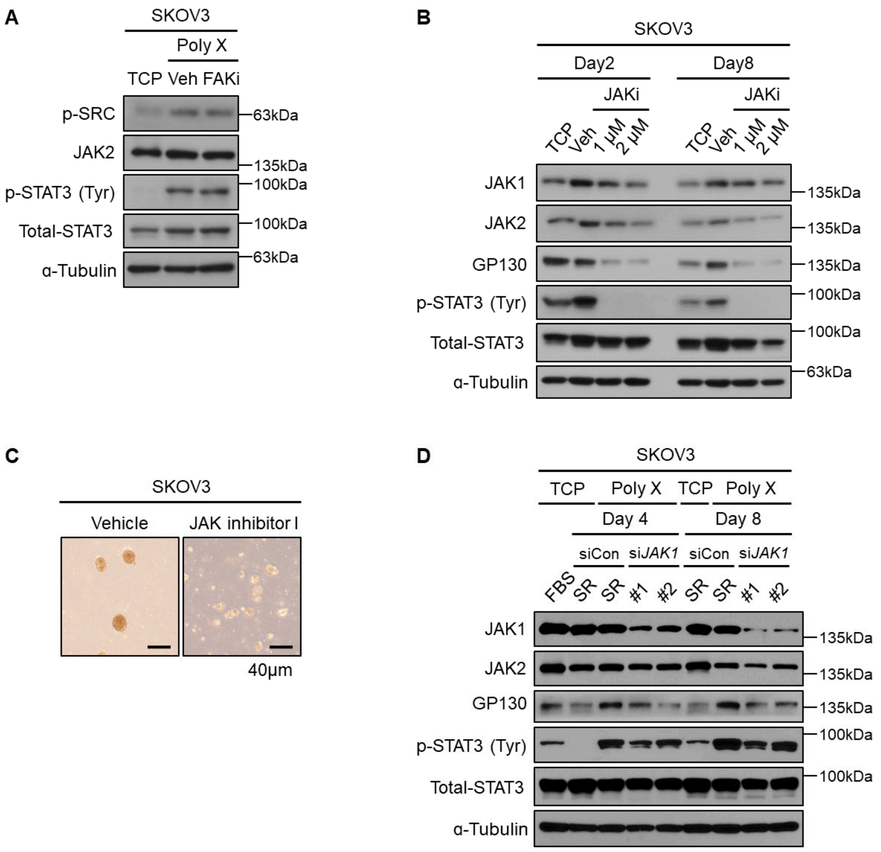

Figure S2. Activation of STAT3 phosphorylation by JAK2 rather than JAK1 in the SKOV3 cells grown on polymer X. (**A**)Cell lysates from SKOV3 cancer cells administered with FAK inhibitor, Y15 cultured on TCP or polymer X for 8 days were immunoblotted with antibodies specific to p-SRC, JAK2, p-

STAT3, total STAT3, and α-tubulin. (**B**)Cell lysates from SKOV3 cancer cells treated with the JAK inhibitor I cultured on TCP or polymer X for 8 days were immunoblotted with antibodies specific to JAK1, JAK2, GP130, p-STAT3, total STAT3, and α-tubulin. (**C**)Image of tumor spheroids cultured on polymer X while administering JAK inhibitor I. Scale bar, 40 μm. (**D**)Cell lysates from SKOV3 cancer cells transfected with either non-target siRNA or si*JAK1*, cultured on polymer X on days 4 and 8 were immunoblotted with antibodies specific to JAK1, JAK2, GP130, p-STAT3, total STAT3, and α-tubulin.

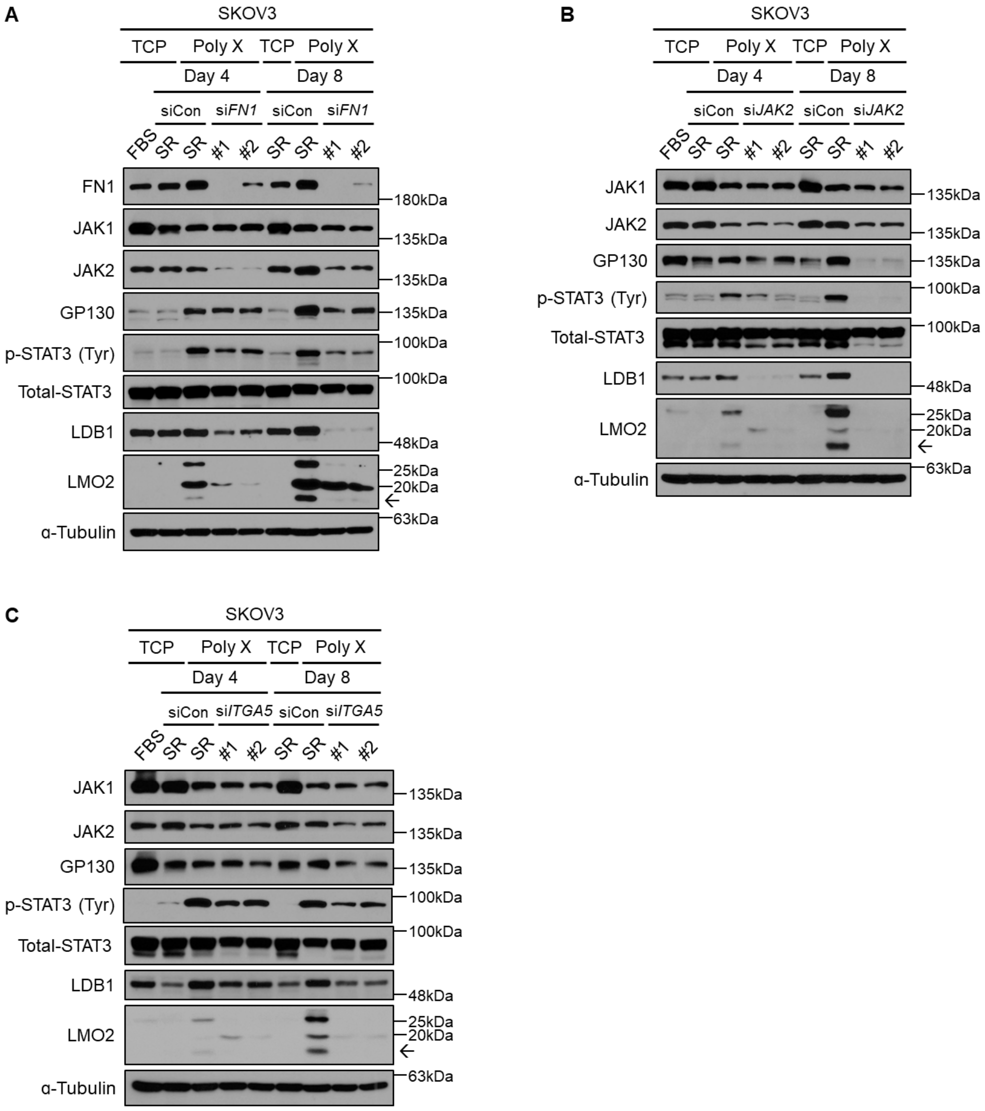

Figure S3. Expression of STAT3 signaling and LMO2/LDB1 proteins in the FN-, JAK2-, or Integrin α5-depleted SKOV3 cells grown under polymer X or TCP culture conditions. (**A**)Cell lysates from SKOV3 cancer cells transfected with either non-target siRNA or si*FN1*, cultured on polymer X on days 4 and 8 were immunoblotted with antibodies specific to FN1, JAK1, JAK2, GP130, p-STAT3, total STAT3, LMO2, LDB1 and α-tubulin. (**B**)Cell lysates from SKOV3 cancer cells transfected with either non-target siRNA or si*JAK2*, cultured on polymer X on days 4 and 8 were immunoblotted with antibodies specific to FN1, JAK1, JAK2, GP130, p-STAT3, total STAT3, LMO2, LDB1, and α-tubulin. (**C**)Cell lysates from SKOV3 cancer cells transfected with either non-target siRNA or si*ITGA5*, cultured on polymer X on days 4 and 8 were

immunoblotted with antibodies specific to FN1, JAK1, JAK2, GP130, p-STAT3, total STAT3, LMO2, LDB1, and α-tubulin.
